# Legionnaires’ disease case-finding algorithm, attack rates, and risk factors during a residential outbreak among older adults: an environmental and cohort study

**DOI:** 10.1186/1471-2334-13-291

**Published:** 2013-06-27

**Authors:** Benjamin J Silk, Jennifer L Foltz, Kompan Ngamsnga, Ellen Brown, Mary Grace Muñoz, Lee M Hampton, Kara Jacobs-Slifka, Natalia A Kozak, J Michael Underwood, John Krick, Tatiana Travis, Olivia Farrow, Barry S Fields, David Blythe, Lauri A Hicks

**Affiliations:** 1 Epidemic Intelligence Service, Office of Workforce and Career Development, U.S. Centers for Disease Control and Prevention, Atlanta, Georgia, USA; 2 Respiratory Diseases Branch, Division of Bacterial Diseases, National Center for Immunization and Respiratory Diseases, U.S. Centers for Disease Control and Prevention, Atlanta, Georgia, USA; 3 Division of Nutrition, Physical Activity, and Obesity, National Center for Chronic Disease Prevention and Health Promotion, U.S. Centers for Disease Control and Prevention, Atlanta, Georgia, USA; 4 Division of Cancer Prevention and Control, National Center for Chronic Disease Prevention and Health Promotion, U.S. Centers for Disease Control and Prevention, Atlanta, Georgia, USA; 5 Baltimore City Health Department, Baltimore, Maryland, USA; 6 Maryland Department of Health and Mental Hygiene, Baltimore, Maryland, USA; 7 College of Human Medicine, Michigan State University, East Lansing, Michigan, USA

**Keywords:** *Legionella*, Community-acquired pneumonia, Elderly

## Abstract

**Background:**

During a Legionnaires’ disease (LD) outbreak, combined epidemiological and environmental investigations were conducted to identify prevention recommendations for facilities where elderly residents live independently but have an increased risk of legionellosis.

**Methods:**

Survey responses (n = 143) were used to calculate attack rates and describe transmission routes by estimating relative risk (RR) and 95% confidence intervals (95% CI). Potable water collected from five apartments of LD patients and three randomly-selected apartments of residents without LD (n = 103 samples) was cultured for *Legionella*.

**Results:**

Eight confirmed LD cases occurred among 171 residents (attack rate = 4.7%); two visitors also developed LD. One case was fatal. The average age of patients was 70 years (range: 62–77). LD risk was lower among residents who reported tub bathing instead of showering (RR = 0.13, 95% CI: 0.02–1.09, P = 0.03). Two respiratory cultures were characterized as *L*. *pneumophila* serogroup 1, monoclonal antibody type Knoxville (1,2,3), sequence type 222. An indistinguishable strain was detected in 31 (74%) of 42 potable water samples.

**Conclusions:**

Managers of elderly-housing facilities and local public health officials should consider developing a *Legionella* prevention plan. When *Legionella* colonization of potable water is detected in these facilities, remediation is indicated to protect residents at higher risk. If LD occurs among residents, exposure reduction, heightened awareness, and clinical surveillance activities should be coordinated among stakeholders. For prompt diagnosis and effective treatment, clinicians should recognize the increased risk and atypical presentation of LD in older adults.

## Background

Legionnaires’ disease (LD) is a potentially-fatal pneumonia caused by inhalation of water aerosols containing *Legionella* bacteria. In the United States, ~8,000-18,000 persons with community-acquired LD are hospitalized annually [1]. Annual incidence rates of LD increase with age to more than ~1.6 cases per 100,000 among persons ≥65 years old [2-4] and LD-related mortality is higher among older adults compared with younger persons [5]. Immunosenescence contributes to the disproportionate burden of LD among the elderly. Also, certain medical conditions known to increase the risk of acquiring LD, including chronic lung disease, lung cancer, hematologic malignancies, end-stage renal disease, diabetes mellitus, and immunosuppression [5], are more prevalent in older persons [6]. Swallowing difficulties, which are more common in the elderly, further increase the risk of lung inoculation through aspiration.

Most LD cases occur sporadically; relatively few patients with LD are diagnosed and reported to public health officials [2]. Therefore, detection of a LD outbreak creates important opportunities for prevention through the elimination of transmission sources and colonization reservoirs in community and residential settings and opportunities for prompt, effective treatment of individuals infected during an outbreak [7,8]. LD outbreaks among residents of long-term care facilities and nursing homes have been publicized and described elsewhere [9]. However, few published reports have described LD outbreaks in apartment buildings and other residential facilities designed for seniors who live independently, even though these facilities are increasingly common in the United States [10,11].

On October 15, 2009, the Baltimore City Health Department and the Maryland Department of Health and Mental Hygiene contacted the Centers for Disease Control and Prevention (CDC) regarding an outbreak of LD among residents of an apartment complex for seniors in Baltimore. In the previous 5 weeks, four residents and a visitor to the complex had been hospitalized with LD. One patient had died and another was receiving intensive care. We investigated to find additional cases, identify exposures associated with disease transmission, determine the source of *Legionella* colonization (if any) at the complex, and recommend interventions to prevent additional cases.

## Methods

Epidemiological and environmental investigations were conducted at the apartment complex, which consisted of four adjacent buildings. Two buildings were built in 2004; the third and fourth buildings were built in 2006 and 2009, respectively. Occupancy of the buildings began soon after construction of each was complete. An age of 62 years or older was required for residency. Single residents occupied most (>90%) of the 340 apartment units at the complex and virtually all units were occupied by October, 2009. Common areas were available to residents (e.g., communal kitchens, laundry facilities, and public restrooms). Medical services were not available onsite, but communal meals and social gatherings occurred regularly.

For case finding, we defined confirmed cases of LD as a diagnosis of clinical or radiographic pneumonia and laboratory-confirmation of LD (i.e., urine antigen testing, culture identification of *Legionella* from respiratory secretions, or a four-fold increase in serum antibody titer to *L*. *pneumophila* serogroup 1) between August 1, 2009 and December 31, 2009 among a resident of any apartment building in the complex. Possible cases were defined similarly, but had either incomplete (i.e., a single *Legionella* titer) or no specific laboratory testing for *Legionella* infection performed. Respiratory illnesses in persons with negative *Legionella* testing were considered non-cases.

**Figure 1:**
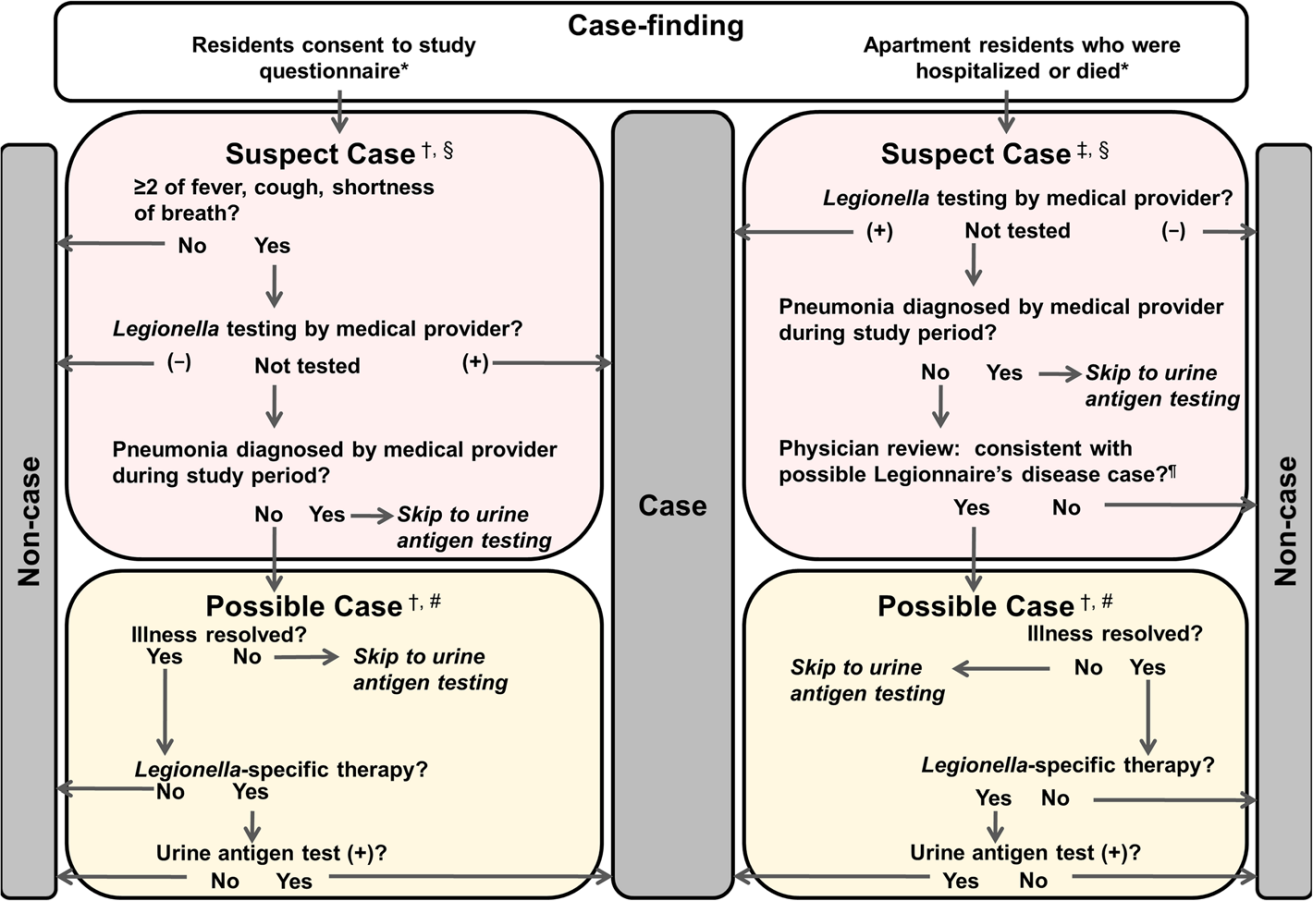
**Decision algorithm for Legionnaire’s disease case-finding and classification.** * The decision courses based on interview data (left) and medical records (right) are not necessarily mutually exclusive. Information for some subjects was gathered in both tracts. ^†^ Data from patient interview. ^‡^ Data from medical records review ^§^ Suspect cases: a resident identified by case-finding who has entered the decision algorithm because his/her illness is to be evaluated as a case of Legionnaire’s disease; resident’s illness remains a suspect case until further categorized. ^¶^ Physicians reviewed available medical records for signs, symptoms, and diagnostic testing (e.g., chest x-ray) to determine if subject possibly had pneumonia consistent with Legionnaire’s disease. ^#^ Possible case: a subject whose illness has met all the criteria of a suspect case is labeled a possible case while outstanding information or testing is collected.

We initiated enhanced surveillance for LD using four strategies. First, we sent email alerts to local hospital infection preventionists to inform them of the outbreak, encourage *Legionella*-specific laboratory testing in patients with community-acquired pneumonia, and remind them to report LD cases. Second, we requested lists of all laboratory-confirmed cases of LD since August 1 from the city’s 12 largest hospitals. Third, we developed a decision algorithm (Figure 1) to identify suspect cases and standardize case classification. During the cohort study (described below), we queried residents for three symptoms (fever, cough and shortness of breath) since August 1, 2009. If the resident had any two of these symptoms, a standardized interview followed. We offered urine antigen testing to individuals with suspected pneumonia who had not had *Legionella*-specific testing; tests were conducted at the Maryland Department of Health and Mental Hygiene’s public health laboratory. Two investigator-physicians reviewed and classified medical records of patients with suspected LD using a standardized data abstraction instrument. Fourth, residents' death certificates were also reviewed for diagnoses of “pneumonia” and “Legionnaires’ disease”.

To estimate attack rates and identify transmission routes, we conducted a retrospective cohort study among all 171 residents of the two affected buildings where LD cases had occurred (A-1 and A-2). The exposure period for the study was defined as August 1 through the date of survey interview. After informed consent was obtained, a standardized questionnaire was administered in person. We queried respondents about their demographic characteristics, water exposures at the complex and within the community, signs or symptoms of respiratory illness, healthcare use and diagnoses, smoking, and comorbid conditions (asthma; chronic lung, kidney, and liver disease; diabetes mellitus; heart disease or congestive heart failure; plus, immunocomprising conditions, including cancer, immunosuppressive therapies, human immunodeficiency virus infection, and organ transplant). Complaints about municipal water service interruptions and water quality (i.e., taste, smell, and appearance) were also recorded. We made at least two attempts on separate days to interview each resident; visitors were not included in the cohort study.

We maintained data in a *Microsoft Access* database (Microsoft Corp, Redmond, WA) and analyzed it in *SAS* version 9.2 (SAS Institute, Inc., Cary, NC). Attack rates (AR) for specific transmission risk factors (present vs. absent) were compared to calculate relative risks (RR) with 95% confidence intervals (95% CIs) and Fisher’s exact tests. A p-value less than 0.10 was considered statistically significant because of the small number of LD cases.

Our environmental investigation began with a physical inspection, noting the orientation of the apartment buildings, the design of the individual apartment units, and points of water use (e.g., showerheads, sink faucets) in each unit. Common areas where water exposures might have occurred were also noted. Diagrams of the potable water system were not available at this stage of the investigation.

On October 17, we collected 103 samples from five apartments of LD patients in affected buildings (A-1 and A-2) and three randomly-selected apartments of residents who had not developed LD in A-2 and each unaffected buildings (U-1 and U-2). (A resident of a randomly-selected apartment in A-1 developed LD after the apartment was sampled.) Specific sampling locations in the apartments included showerheads, sink faucets in kitchens and bathrooms, and water heater tanks (n = 56 samples). We also collected samples from outdoor fire hydrants (n = 6), from the buildings’ water intake valves (n = 19), and the sink faucets and water heating tanks of common areas in buildings A-1 and A-2 (n = 22). We collected samples of municipal water and expansion tanks, designed to absorb excess water pressure caused by thermal expansion or “water hammers” (i.e., water pressure surges), on October 21 and October 24, respectively. Biofilm swabs and one-liter bulk water samples were collected according to published procedures [12]. Water temperatures and free chlorine concentrations (using N,N-diethyl-P-phenylenediamine reagents) were measured in potable water systems during this sampling.

At the CDC *Legionella* laboratory, bulk water samples were filtered through polycarbonate 0.2 μm filters. The filters were placed in 5 ml of sterile water and vortexed for 60 seconds. One hundred μl of this suspension were placed on buffered charcoal-yeast extract media with and without antibiotics. Plates were incubated at 95–98.6°F (35–37°C) in 2.5% CO_2_ to culture *Legionella*. We also forwarded two patients’ clinical isolates to CDC.

Serogrouping and determination of monoclonal antibody patterns [13,14] were performed on all isolates. In addition, sequence-based typing was used to create seven-gene profiles (flaA, pilE, asd, mip, mompS, proA, and neuA) of the clinical isolates and select environmental isolates [15-17]. Sequence-based typing results were compared for clinical and environmental isolates to determine the source of the outbreak strain in the environment.

This study was determined an emergency public health investigation exempt from institutional review board (IRB) review; standard procedures to protect patient confidentiality were followed.

## Results

### Demographics of residents and patients

The demographics of residents and LD patients were similar. Almost all of the 352 residents were either African-American (91%) or Korean-American (7%). Three-quarters (75%) of residents were female. Residents varied in age (62–69 years: 26%, 70–79: 45%, 80–89: 26%, 90 and older: 3%). In comparison, all eight residents who developed LD between September and December of 2009 were African-American. Six (75%) were female. Among residents, patients were younger than non-patients, but this difference was not significant (p = 0.09) (Table 1). Two younger visitors also acquired LD.

**Table 1:** Characteristics of Legionnaires’ disease patients among residents and visitors of an apartment complex for seniors

**Patient**	**Building**	**Diagnostic**	**Onset Month**	Age group*	**Outcome**
1	A-2	Urine antigen	September	70-79	Recovered
2	A-2	Urine antigen	September	62-69	Recovered
3	A-2	Urine antigen	September	70-79	Died
4^**^	A-2	Urine antigen	October	<62	Recovered
5	A-1	Urine antigen	October	62-69	Recovered
6	A-1	Sputum culture	October	62-69	Recovered
7	A-1	Sputum culture	October	70-79	Recovered
8	A-1	Urine antigen	October	70-79	Recovered
9	A-1	Urine antigen	October	62-69	Recovered
10^**^	A-2	Urine antigen	December	<62	Recovered

^*^ For comparison, the frequency distribution of age group among all residents of the apartment complex was 62–69 years old: 26%, 70–79: 45%, 80–89: 26%, and 90 and older: 3% (p = 0.09).

^**^ Visitors to the apartment complex.

### Exposures and disease transmission

Most (80%) cases were identified by urine antigen testing performed during hospitalization, with the exception of one case that was detected retrospectively ~47 days after illness onset (Figure 1). LD occurred among residents of buildings A-1 and A-2; no LD cases were detected among residents or visitors of the two other buildings (U-1 or U-2). Cohort study participation was high among residents of A-1 (86%) and A-2 (91%) (n = 143 respondents).

The eight LD cases that occurred among 171 residents of A-1 and A-2 represented an attack rate of 4.7% (Table 2). Although not statistically significant, risk of LD appeared to be higher among persons who reported current smoking (RR = 3.02, 95% CI: 0.67–13.58, P = 0.14) or the presence of an immunocompromising condition (RR = 3.33, 95% CI: 0.71–15.70, P = 0.12) compared with residents without these risk factors. All eight LD patients reported showering, including one patient who also reported bathing in a tub. Tub baths were protective against disease transmission in the cohort (RR = 0.13, 95% CI: 0.02–1.09, P = 0.03). Attack rates among the 40 respondents who reported showering at least daily (n = 3 patients, AR = 7.5%) and 53 respondents who showered less often (n = 3 patients, AR = 5.7%) were similar (P = 0.70).

**Table 2:** Legionnaires’ disease attack rates and relative risks among residents of an apartment complex for seniors (n = 143 respondents)

**Characteristics**	**No. of LD patients**	**No. of non-patients**	**Attack rate**	Relative risk^* ^**(95% confidence interval)**
**Overall**	8	135	4.7%^†^	--
**Potable water exposures**				
Any showering	8	90	8.2%	7.02 (0.45–128.21)
Any tub baths	1	70	1.4%	0.13 (0.02–1.09)^‡^
**Smoking**				
Current	2	12	14.3%	3.02 (0.67–13.58)
Former (>100 cigarettes)	3	57	5.0%	1.07 (0.22–5.08)
**Comorbid condition**				
Asthma	0	16	0%	0.48 (0.03–7.10)
Chronic lung disease	0	9	0%	1.02 (0.07–18.76)
Chronic kidney disease	0	11	0%	0.74 (0.04–10.46)
Diabetes mellitus	1	51	1.9%	0.45 (0.03–1.93)
Heart disease	1	35	2.8%	0.42 (0.05–3.27)
Immunocompromised^§^	2	13	13.3%	3.33 (0.71– 15.70)

^*^ Relative risk (RR) compares attack rates for persons with and without the exposure or risk factor. RRs were estimated by adding 0.5 to cells with zero values (asthma, and chronic lung or kidney disease).

^†^ Overall attack rate based on all residents (n = 171), including non-respondents.

^‡^ P < 0. 05.

^§^ Immunosuppressive therapy, human immunodeficiency virus (HIV) infection, or organ transplant.

Although not included in the cohort, the two visitors who developed LD reported intensive exposures to water either during dishwashing (patient 4) or while cleaning the kitchen and bathroom using a bucket filled with water (patient 10) (Table 1); neither showered or bathed in the apartments. None of the residents with LD reported other known exposures to aerosolized water outside the apartment complex.

### Environmental factors favoring colonization

The entire complex was served by the same municipal water system. Free chlorine residual was not detected in any bulk samples collected from showerheads or sink faucets during pre-remediation sampling. Each apartment had its own water heating tank. Free chlorine concentrations of 0.1 mg/dL were detected in three of the eight water heating tanks in patient and non-patient apartments. All water heaters had a factory temperature setting of ~120° F (~48.9°C), but temperature differences were observed in water collected from faucets at the bottom of tanks in sampled apartments (range: 70–100°F/21.1–37.8°C).

Three-quarters (75%) of residents who were interviewed noted at least one interruption in water service since August 1^st^. More than half of residents (56%) reported changes in the taste, odor, or color of their water during the same period.

### Outbreak strain

Clinical isolates of *L*. *pneumophila* from patients 6 and 7 were characterized as *L*. *pneumophila* serogroup 1, monoclonal antibody type Knoxville (1,2,3). The outbreak strain was determined to be sequence type 222 (i.e., allelic profile 2, 19, 5, 10, 18, 1, 10).

Among the 103 pre-remediation samples collected, *L*. *pneumophila* 1 (1,2,3) was exclusively detected in apartments within buildings A-1 and A-2 where all LD patients resided (Table 3). Specifically, the outbreak strain was detected in 18 (86%) of 21 samples collected from three patients’ apartments in A-1 and in 13 (62%) of 21 samples from two patients’ apartments and one non-patient apartment in A-2. Sequence type 222 was isolated from at least one source in all five patients’ apartments where samples were collected, including water heaters in the apartments of patients 1 and 6, the kitchen sink faucet of patient 3 (whose son, patient 4, reported washing dishes and also experienced LD), the bathroom sink faucet of patient 5, and the shower and water heating tank in the apartment of patient 7. Although *Legionella* was detected in samples of water intake valves and common areas of buildings A-1 and A-2, none of the isolates were *L*. *pneumophila* 1 (1,2,3).

**Table 3:** ***Legionella *culturing and monoclonal antibody testing of potable water samples collected from an apartment complex for seniors (n = 103 samples)**

**Sample site**	**No. tested**	**No. (%) positive**	
		Any Legionella*	*L. pneumophila *1 (1,2,3)**
**Affected buildings (A-1 and A-2)**^†^			
Water intake valves	11	6 (55%)	0
A-1 apartments	21	21 (100%)	18 (86%)
A-2 apartments	21	20 (95%)	13 (62%)
Common areas	22	12 (55%)	0
**Unaffected buildings (U-1 and U-2)**^†^			
Water intake valves	8	1 (13%)	0
U-1 apartments	7	4 (57%)	0
U-2 apartments	7	6 (86%)	0
**Fire hydrants**	6	2 (33%)	0

* Any *Legionella* includes *L. pneumophila* and other undetermined species of blue-white *Legionella* that typically are not pathogenic.

**L. pneumophila 1 (1,2,3) denotes serogroup 1, monoclonal antibody type Knoxville (1,2,3).

^†^ Cases occurred among residents and visitors of affected buildings A-1 and A-2 only.

## Discussion

A large LD outbreak occurred at an apartment complex for seniors in Baltimore between September and December, 2009. Ten cases were confirmed among eight residents and two visitors to the complex; one case was fatal. *Legionella* respiratory cultures were available from two patients and characterized as *L*. *pneumophila* serogroup 1, monoclonal antibody type Knoxville (1,2,3), sequence type 222. An indistinguishable strain was detected in numerous potable water sources from apartment units of two buildings. Epidemiological data from a cohort study with a high response rate indicated that all patients were primarily or exclusively exposed to colonized potable water in apartments. Taken together, the environmental and cohort studies implicated *Legionella* colonization of the potable water system as the source of the outbreak.

The convergence of several factors led to the outbreak. Residents of the apartment complex were particularly susceptible to LD because of their advanced age, high prevalence of underlying conditions, and smoking habits. Although the size of the cohort limited statistical power, we confirmed that attack rates were highest in persons who smoked or had an immunocompromising condition. However, two younger visitors (including one without underlying risk factors) also developed LD. This finding suggested that the type, frequency, and duration of exposures may also determine disease risk. Also, the potable water system’s conditions were conducive to *Legionella* growth. Chlorine disinfection was not detected at the point of use in any of the residents’ apartments; hot water temperatures were generally within the ideal range for *Legionella* amplification (77°F-108°F/25°C-42.2°F). Importantly, the potable water system was colonized with a monoclonal antibody 2 positive [18], a virulent strain of *L*. *pneumophila* (sequence type 222) that has been previously associated with outbreaks in the northeastern United States and Canada [19,20]. Nevertheless, the root cause of the outbreak was not determined conclusively during the outbreak investigation.

Increases in the burden of LD [4] may be related to the expanding population of older adults in the United States. Given the fact that a substantial number of residential facilities for the elderly exist [10,11], facility managers, healthcare providers, caregivers, and local public health officials need to be aware of the potential for outbreaks among this population at higher risk for LD and LD-related mortality. Despite several published reports of LD outbreaks in nursing homes [9,21,22], this is the first published report of an outbreak at an apartment complex designed for elderly residents who live relatively independently.

Facility managers should consider developing a *Legionella* prevention plan in coordination with local public health officials as well as plumbers, engineers, or other environmental consultants. This plan may involve completing an environmental assessment to identify opportunities to reduce colonization risk [23]. (A sample environmental assessment plan can be found here: http://www.cdc.gov/legionella/index.html.) Hazard analyses and critical control points (HACCP), an industry method designed to improve food safety, can be applied to *Legionella* prevention – as outlined by forthcoming practices from the American Society of Heating, Refrigerating and Air-Conditioning Engineers, Inc. (ASHRAE) Standard Project Committee 188 (http://spc188.ashraepcs.org/). Facility managers may also consider periodic environmental monitoring of the potable water system to identify *Legionella* colonization in the context of a full HACCP plan. When possible, potable water systems should be designed to minimize the potential for *Legionella* colonization (e.g., short, direct pipes with appropriate insulation and materials).

In facilities known to be colonized, remediation measures to protect vulnerable residents are indicated and often involve halogen-based disinfectants, such as chlorine or chlorine dioxide [24-26]. Alternatively, permanent elevation of water heater temperatures (>130°F/ >54.4°C) can reduce *Legionella* viability [27], but concerns for scalding among older individuals [28] may prevent this approach from being a feasible option without safeguards (e.g., thermostatic mixing valves). Facilities with *Legionella* colonization should also adhere to the ASHRAE guidelines for minimizing legionellosis risk [23].

If LD cases have been detected among residents, exposure reduction, heightened awareness, and clinical surveillance [9] should be accomplished through coordination among facility managers, healthcare providers, caregivers, and public health officials. To reduce exposure when potable water is implicated, we found that bathing in a tub appeared to be protective. Because some residents may have bathed in a tub and showered periodically, we may have underestimated the true protective effect of exclusive tub bathing. The finding that tub bathing is protective is plausible because it can reduce continuous exposure to water aerosols, which would otherwise be experienced while showering. Other measures to minimize contact with aerosolized potable water, including exposure reductions during dishwashing or cleaning of kitchens, bathrooms, or other areas, also should be evaluated and promoted. If a dishwasher is unavailable, for example, a sink could be filled with running water while positioning oneself a distance away, as an alternative habit that may be effective in avoiding repeated inhalation of aerosolized water when dishes are washed individually. To heighten awareness for early detection of LD, information on signs and symptoms of acute respiratory disease and pneumonia, and recommendations for immediate healthcare-seeking, should be provided to residents and family members as well as medical directors, nurses, infection control professionals, and other caregivers. Added effort may be required to communicate effectively among older adults with varying degrees of health literacy, dementia, and other challenges. Socioeconomic, ethnic/cultural, and language barriers may also need to be recognized. Finally, clinical surveillance can be accomplished through specific diagnostic testing and systematic monitoring for symptoms of acute illness. Urinary antigen testing is indicated for suspected *L*. *pneumophila* serogroup 1 infections, which represent the majority (~84%) of LD cases in the United States [29]. Caregivers should also promptly recognize and refer residents with acute, febrile respiratory illness to healthcare providers.

The clinical presentation of LD pneumonia can be atypical, particularly among elderly patients [6,30]. Therefore, a heightened index of suspicion among clinicians and healthcare providers is also indicated to accelerate diagnosis and effective treatment of *Legionella* pneumonia among residents and visitors to facilities with colonization or the occurrence of LD cases. In addition to *Legionella* urine antigen testing, clinicians should attempt to recover respiratory secretions for *Legionella* culture from any patient linked to an outbreak and any patient hospitalized with severe community-acquired pneumonia of unknown etiology because clinical *Legionella* isolates are critical for establishing an association with an environmental exposure and can direct appropriate antimicrobial therapy (e.g., levofloxacin). Healthcare providers should also work closely with patients to eliminate modifiable risk factors for pneumonia (e.g. smoking) [5], optimize the management of underlying medical conditions (e.g., asthma, diabetes mellitus), and educate patients on behaviors that reduce exposures to aerosolized water.

## Conclusions

Managers of elderly-housing facilities, local public health officials, and other stakeholders should implement *Legionella* prevention and control strategies based on whether *Legionella* colonization of potable water has been detected and especially when LD cases occur. Physicians specializing in infectious diseases and other persons providing healthcare services to elderly residents should be aware of the increased risk of LD in this population; awareness is a prerequisite for the provision of quality healthcare, including prompt diagnosis and effective treatment as well as counseling on the importance of risk modification and exposure reduction to prevent additional morbidity and mortality.

## Competing interests

The authors declare that they have no competing interests. The findings and conclusions in this article are those of the authors and do not necessarily represent the official position of U.S. Center for Disease Control and Prevention.

## Authors' contributions

BJS, JLF, LMH, KJS, OF, DB, and LAH lead the investigation, including design of the environmental and cohort studies. BJS, JLF, KN, MGM, LMH, KJS, JMU, JK, and DB collected environmental and epidemiological data. EB, NAK, TT, and BSF conducted laboratory analyses. BJS, JLF, and LAH drafted the manuscript. All authors read and approved the final manuscript.

## Pre-publication history

The pre-publication history for this paper can be accessed here:

http://www.biomedcentral.com/1471-2334/13/291/prepub
